# miR319-Regulated TCP3 Modulates Silique Development Associated with Seed Shattering in Brassicaceae

**DOI:** 10.3390/cells11193096

**Published:** 2022-10-01

**Authors:** Biting Cao, Hongfeng Wang, Jinjuan Bai, Xuan Wang, Xiaorong Li, Yanfeng Zhang, Suxin Yang, Yuke He, Xiang Yu

**Affiliations:** 1 School of Life Sciences and Biotechnology, Shanghai Jiao Tong University, Shanghai 200240, China; 2 Shanghai Key Lab of Protected Horticultural Technology, Horticultural Research Institute, Shanghai Academy of Agricultural Sciences, Shanghai 201106, China; 3 National Key Laboratory of Plant Molecular Genetics, Center for Excellence in Molecular Plant Science, Shanghai Institute of Plant Physiology and Ecology, Chinese Academy of Sciences, Fenglin Road 300, Shanghai 200032, China; 4 The Key Laboratory of Plant Development and Environmental Adaptation Biology, Ministry of Education, School of Life Science, Shandong University, Qingdao 266101, China; 5 Hybrid Rape Research Center of Shaanxi Province, Yangling 712100, China; 6 Key Laboratory of Soybean Molecular Design Breeding, Northeast Institute of Geography and Agroecology, Changchun 130102, China

**Keywords:** TCP3, miR319, Arabidopsis, rapeseed, silique shattering

## Abstract

Seed shattering is an undesirable trait that leads to crop yield loss. Improving silique resistance to shattering is critical for grain and oil crops. In this study, we found that miR319-targeted TEOSINTE BRANCHED 1, CYCLOIDEA, and PROLIFERATING CELL NUCLEAR ANTIGEN BINDING FACTOR (TCPs) inhibited the process of post-fertilized fruits (silique) elongation and dehiscence via regulation of FRUITFULL (FUL) expression in Arabidopsis *thaliana* and *Brassica napus*. AtMIR319a activation resulted in a longer silique with thickened and lignified replum, whereas overexpression of an miR319a-resistant version of AtTCP3 (mTCP3) led to a short silique with narrow and less lignified replum. Further genetic and expressional analysis suggested that FUL acted downstream of TCP3 to negatively regulate silique development. Moreover, hyper-activation of BnTCP3.A8, a *B. napus* homolog of AtTCP3, in rapeseed resulted in an enhanced silique resistance to shattering due to attenuated replum development. Taken together, our findings advance our knowledge of TCP-regulated silique development and provide a potential target for genetic manipulation to reduce silique shattering in Brassica crops.

## 1. Introduction

Angiosperms show various types of siliques, most of which are derived from the ovary walls and fertilized ovules. Their structures are conserved in *Arabidopsis thaliana* and some species of the Brassicaceae family, including cabbage, broccoli, Chinese cabbage (*Brassica rapa*), and rapeseed (*Brassica napus*) [1]. In *Arabidopsis thaliana*, siliques are composed of fertilized ovules and three major regions, namely the valves, repla, and valve margins. The valve margins were formed between the valves and the repla, and two types of cells at the valve margins regulated the silique opening. The separation layer is also called dehiscence zone (DZ), which is composed of 2–3 layers of thin-walled cells and separates the heavily lignified cells of the pericarp edge from the replum. Fruit dehiscence is accompanied by degradation of the thin-walled cells [2,3,4,5]. Canola seeds, which are harvested for oil, are often lost owing to premature silique shattering, particularly under adverse weather conditions, and premature dehiscence or silique shattering leads to significant crop losses [6]. Reducing silique shattering will increase the proportion of seed obtained during harvest, which is conducive to economic income.

Several key genes contributing to silique development have been identified in *Arabidopsis*. *REPLUMLESS* (*RPL*) plays an important role in replum development. *rpl* mutants exhibit reduced replum width, and the strong alleles display a complete absence of outer replum [7]. *SHATTERPROOF1* (*SHP1*) and *SHP2* are necessary for the proper valve margins development. The loss of *SHP1* and *SHP2* function results in lacking the lignified and separation layers in siliques, which prevents silique opening [8]. *INDEHISCENT* (*IND*) and *ALCATRAZ* (*ALC*) work downstream of *SHP* [9,10]. The atypical bHLH protein IND is required for seed dissemination [9]. The small cells of the separation zone and the adjacent lignified cell layers were defective in the siliques of the *ind* mutant, and mutation of *ALC* resulted in the lack of the non-lignified cell layer in the separation layer. *NO TRANSMITTING TRACT* (*NTT*) encodes a zinc finger transcription factor, and loss of *NTT* function affects the normal transmitting-tract development [11] and replum development in *Arabidopsis* fruits [12]. In addition, the siliques of activation-tagged allele of *NTT* (ntt-3D) are indehiscence and almost lack separation and lignification layer [13]. The cells in the mesophyll tissue layer of *fruitfull* (*ful*) mutant are lignified at the later stage of fruit development and are much smaller than in the wild type [14,15]. Overall, the FUL-SHP network play important role in fruit morphology, which is evolutionally conserved in plants [16].

*Brassica* species are the most closely related crops to *Arabidopsis*, and they disperse seeds in a similar way [17,18]. Ectopic expression of the *Arabidopsis AtFUL* gene under control of the Cauliflower Mosaic Virus 35S promoter in *Brassica juncea* leads to complete shattering-resistant siliques similar to those of *35S::FUL* transgenic plants [19,20]. *JAGGED* (*JAG*) is involved in maintaining the integrity of boundaries between cell groups with indeterminate or determinate fates. *BnJAG-33* mutants enhance silique shatter resistance [21]. *BraA.IND.a* and *BolC.IND.a* genes control valve margin cell fate and inhibit replum formation [22]. *BnIND* mutations in *B. napus* show higher shatter resistance [23,24]. Taken together, these conserved genetic regulators of silique development can be applied in *Brassica* breeding for improving seed shattering resistance.

The plant-specific TEOSINTE BRANCHED 1, CYCLOIDEA, and PROLIFERATING CELL NUCLEAR ANTIGEN BINDING FACTOR (TCP) family with a bHLH motif that allows DNA binding and protein–protein interactions are involved in growth-related progress, such as branching, floral organ morphogenesis and leaf development [25,26]. There are 24 members of the TCP family in *Arabidopsis thaliana*, and *TCP2, TCP3, TCP4, TCP10,* and *TCP24* are the targets of miR319 [27]. miR319-regulated TCP genes function in leaf development by regulating cell division arrest [28,29]. In a previous work, we reported that an anther with four microsporangia was transformed into the one with two microsporangia in the *jaw-D* mutant in which the *MIR319a* gene is activated [30]. However, the role of miR319-regulated TCPs in fruit development remains unclear. In this study, we revealed that the siliques replum of *jaw-D* plants, in which the MIR319a gene was activated, was significantly enlarged, and *AtTCP3* was one of the major regulators in against to silique elongation and dehiscence via increasing FUL expression. Finally, hyper-activation of *BnTCP3.A8* in rapeseed increased silique resistance to shattering. These results uncovered that miR319-regulated TCPs played viral role in silique development and can be used as a genetic editing resource for seed shattering resistance in Brassica crops.

## 2. Materials and Methods

### 2.1. Plant Growth Conditions

The *Arabidopsis thaliana* Col-0 ecotype was used as the wild type in this study. Seeds of Col-0, *jaw-D* mutants and transgenic *Arabidopsis* lines were sterilized using 70% (*v*/*v*) ethanol. plants were grown on Murashige and Skoog (MS) plates with 1% sucrose under long-day conditions (16-h light/8-h dark) and then transferred to a growth room at 22 °C under long-day conditions.

The rapeseed accession K407 was acquired from Hybrid Rape Research Center of Shaanxi Province [31]. Rapeseed wild-type (accession K407) and transgenic rapeseed lines overexpressing *BnTCP3.A8* were sown in a greenhouse. Then, the seedlings were transplanted into a field at the Songjiang Farm Station of the Shanghai Institute of Plant Physiology and Ecology in early September.

### 2.2. Plasmid Construction and Transformation

To generate the *mAtTCP3*-overexpression construct, the full-length CDS of *AtTCP3* (AT1G53230) was amplified using specific primers. Then, a silent mutation was introduced into the miR319 target site of the *AtTCP3* CDS to generate the *mAtTCP3* construct using a Site-Directed Mutagenesis Kit (TaKaRa). The chimeric *pAtTCP3::AtTCP3SRDX* was constructed as described previously [32]. To generate the *p35S::AtFUL* construct, the full length CDS of the *AtFUL* gene was amplified. To generate the *p35S::BnTCP3.A8* construct, the full length CDS of the *Brassica napus TCP3* gene (Acc. number GSBRNA2T00114181001) was amplified and cloned into the binary vector pChimera. The *Agrobacterium tumefaciens* strain GV3101 pMP90RK was used for stable plant transformations. All the primers used for plasmid construction are listed in Table S1.

For rapeseed hypocotyl transformations, we followed the protocol described by Liu et al. and Moloney et al. [33,34]. The *p35S::mAtTCP3*, *pAtTCP3:: AtTCP3SRDX* and *p35S:: AtFUL* vectors were transferred into Col-0 using the floral dip method [35]. The *p35S::BnTCP3.A8* and *p35S::mAtTCP3* transgenic plants were selected on MS medium containing 50 mg/mL kanamycin after *Agrobacterium*-mediated transformation. Positive seedlings (T0) were transplanted into soils. In the F1 generation, all transgenic plants were confirmed using PCR with gene-specific primers and maintained in a growth room at 22 °C under long-day conditions. Primers used in this study are listed in Supplementary Table S1.

### 2.3. Isolation of RNA and Real-Time PCR Analysis

Total RNA was extracted from plants using TRIzol reagent. Before performing quantitative real-time PCR, RNA was treated with DNase I (TaKaRa) to avoid DNA contamination, and then RNA was purified with the phenol chloroform. Real-time detection of all target genes was based on the method of Li et al. (2020) [36]. The quantification of the relative expression levels was performed as reported previously [37]. The relative transcript levels of each gene in *Arabidopsis thaliana* and *B. napus* were normalized to that of *Actin* [36] and *UBC21* [38] for quantitation, respectively. Primer Premier 5 software was used to design oligonucleotide primers (http://www.premibiosoft.com (accessed on 1 September 2022)) for real-time PCR to amplify the reference and target genes. All the primers used in this study are shown in Table S1.

### 2.4. Histological Analyses

For toluidine blue staining, the siliques were harvested one week after opening of flower buds and fixed in FAA for more than 18 h, sectioned and stained with 0.1% toluidine blue [7,8,39]. For phloroglucinol lignin staining, the siliques at the same stage were stained with 2% phloroglucinol solution overnight. Then, the siliques were decolorized in ethanol and photographed.

### 2.5. In Situ Hybridization

Silique (stage 10–12) sections in the wild-type flowers were prepared using previously described pretreatment and hybridization methods [40]. The primers used to generate hybridization probes specific for *AtTCP3* using the fragments (513 bp) in coding sequences according to the method of Wang et al. (2015) [30]. LNA (Locked Nucleic Acid) modified probes specific for *AtTCP3* were synthesized and labeled with DIG at the 3′-end by TaKaRa.

### 2.6. Scanning Electron Microscopy (SEM)

Siliques (stage 17) of *Arabidopsis* and *B. napus* were harvested and fixed overnight at 25 °C in FAA, dehydrated through an ethanol series, and critical-point dried. Samples were sputter-coated with gold and viewed using a Hitachi S-2300 electron microscope. Replum width were measured in the middle of siliques photographed according to the method of Marsch-Martinez, N. et al. (2014) [12].

### 2.7. Shattering-Resistance Measurements

Mature siliques were collected for the measurement of silique shattering resistance by a random impact test (RIT) [41]. In total, 20 siliques were preserved in a mesh bag overnight after incubation at 60 °C for 60 min to equilibrate the moisture content, and then, they were placed into a cylindrical container, having a diameter of 6 cm and a height of 17 cm, which was pre-loaded with 12 steel balls with a diameter of 13 mm. On a horizontal shaker, the container was shaken at 300 rpm and then the numbers of cracked siliques were recorded after 1 min shaking intervals. A total of 10 recordings were taken. After each recording, the broken siliques were removed, and all the experiments were performed in triplicate. The silique shattering-resistance index (SRI) was calculated using the following equation:

$$\mathrm{SRI}=1-\sum_{i=1}^{10}Xi\times \left(11-i\right)/200$$

where *Xi* represents the number of cracked siliques in time *i^th^*, with 1 ≤ *i* ≤ 10.

### 2.8. Sequence alignment and Phylogenetic Analysis

Arabidopsis AtTCP protein sequences were downloaded from the Arabidopsis Information Resource (https://www.arabidopsis.org/ (accessed on 1 September 2022)). BnTCP3.A8 and Brp.TCP3 were selected based on their high similarity with AtTCP3. Full-length amino acid sequence multiple alignments were performed using ClustalW and GeneDoc. Unrooted phylogenetic trees were constructed from the aligned amino acid sequences using the neighbor-joining method in MEGA 6.0, and bootstrapping was carried out with 1000 iterations [42].

### 2.9. Statistical Analysis

We calculate statistical significance using two-tailed Student’s *t*-test and error bars indicate standard error (SE). Values of *p* < 0.05 are statistically significant.

## 3. Results

### 3.1. AtMIR319a-Regulated TCPs Played Negative Role in Silique Development

**Figure 1:**
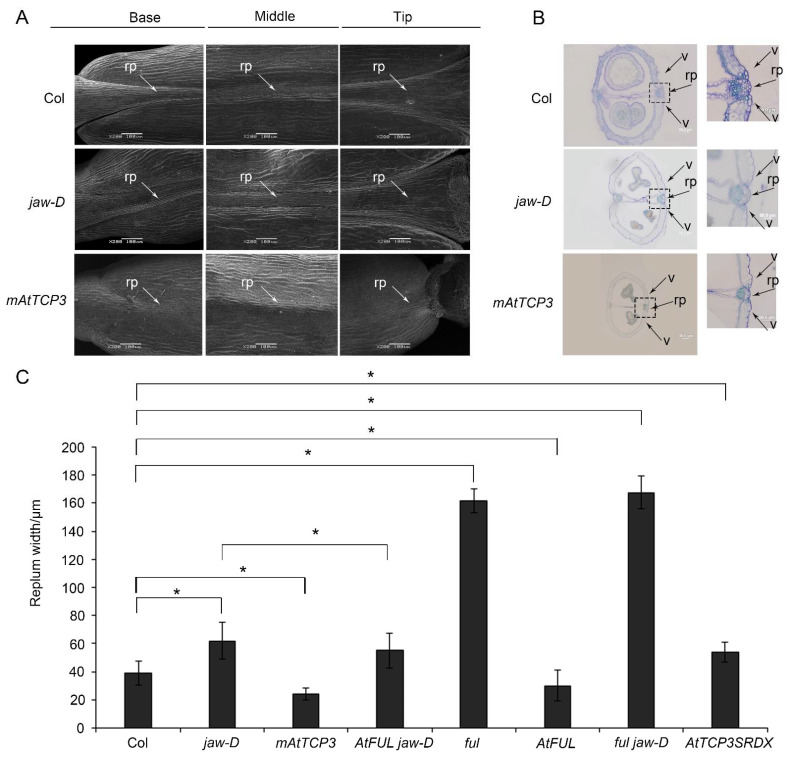
The phenotypes of the repla and dehiscence zones in Col (wild-type), *jaw-D* mutant and *p35S::mAtTCP3* plants. (**A**) Images of scanning electron microscope (SEM) showing the repla of the wild-type, *jaw-D*, and *mAtTCP3* siliques. (**B**) Cross-sections of siliques (stage 17) showing repla and valve margins of the wild-type, *jaw-D*, and *mAtTCP3* siliques in *Arabidopsis*. The repla and valve margins in boxes were magnified in right. (**C**) Bar graph showing replum width of the wild-type (Col), *jaw-D*, *mAtTCP3*, *FUL jaw-D*, *ful*, *AtFUL*, *ful jaw-D*, and *AtTCP3SRDX* siliques. *mAtTCP3*, *p35S::mAtTCP3* lines. *AtFUL*, *p35S::AtFUL* lines. *FUL jaw-D*, *p35S::AtFUL* in *jaw-D* mutant. *ful jaw-D*, double mutants. *AtTCP3SRDX*, *pAtTCP3:: AtTCP3SRDX*. rp, replum. v, valve. * indicates *p* < 0.05.

In previous studies, the development defect of *jaw-D* mutant plant with activation of AtMIR319a was observed in both vegetable and reproductive stages (Wang et al., 2015). However, the molecular mechanism of silique defect in *jaw-D* plant has not been uncovered. Using scanning electron microscopy (SEM), we found that the repla of the *jaw-D* silique was wider than those of the wild type in the base, middle, and tip of the mature silique (Figure 1A). Transverse sections of siliques showed that the *jaw-D* repla were significantly wider compared with wild-type repla (Col-0) in the middle of mature siliques (Figure 1B,C). Thus, these results indicated that activation of AtMIR319a enlarged repla in siliques.

**Figure 2:**
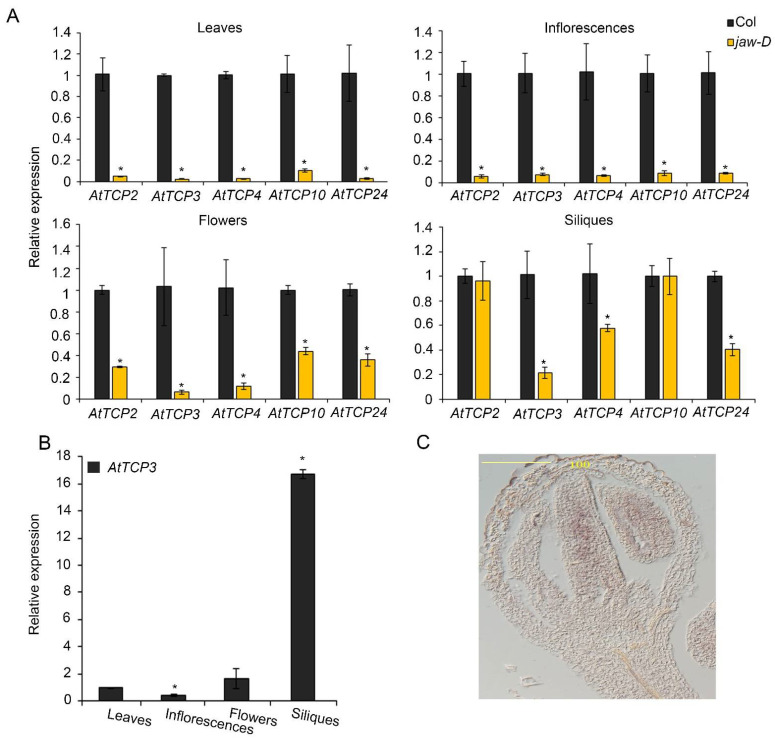
Expression patterns of miR319a-targeted *TCP* genes. (**A**) The relative expression level of *AtTCP* genes in the *jaw-D* mutant cauline leaves, inflorescences, flowers (stage 14–15), and siliques (stage 17) as compared to wild type plants (Col). (**B**) Real-time PCR showing the relative expression levels of *AtTCP3* in cauline leaves, flower buds, flowers, and siliques (stage 17) in wild-type plants (Col). (**C**) In situ hybridization of *AtTCP3* in transverse cross-section of Col siliques (stage 8). Three biological replicates are taken. Error bars indicate SE. * indicates *p* < 0.05.

To investigate the expression changes of the five miR319-targeted *TCP* genes in different tissues of *jaw-D* mutant plants, real-time PCRs were performed to analyze the expression levels of *AtTCP2*, *AtTCP3*, *AtTCP4*, *AtTCP10*, and *AtTCP24* in cauline leaves, inflorescences, flowers, and siliques. In cauline leaves and inflorescences, the expression of *AtTCP2*, *AtTCP3*, *AtTCP4*, *AtTCP10*, and *AtTCP24* were consistently reduced to a similar level in *jaw-D* mutants compared to the wild type (Figure 2A). In contrast, their expressions were differently suppressed in flowers (stage 14–15) and siliques (stage 17). Specifically, *AtTCP3* was mostly suppressed among all the five *TCP* genes in silique of *jaw-D* plants (Figure 2A). Detailed analysis of *AtTCP3* expressional level in different plant tissues confirmed that this gene was most accumulated in siliques as compared to in rosette leaves, inflorescences, and flowers (Figure 2B). Given the siliques of *Arabidopsis thaliana* originated from gynoecium with two fused carpels, we further investigated the mRNA abundance of *AtTCP3* during early flowering stage by in situ hybridization. In young flower buds of wild-type plants, *AtTCP3* transcripts were highly accumulated in the developing carpel (Figure 2C). These results suggested that *AtTCP3* was one of the major regulators of silique development among these five miR319 targeted TCPs.

Notably, *TCP4* and *TCP24* were also significantly repressed in silique of *jaw-D* plants compared to Col-0 (Figure 2A). Knockout mutants of *AtTCP3* showed no visible phenotypic alterations due to the functional redundancy of the miR319-target TCPs [43,44]. To further confirm the function of *AtTCP3* in repla development, we constructed the *pAtTCP3::AtTCP3SRDX* transgenic line fusing TCP3 with plant-specific ERF-associated amphiphilic repression (EAR) motif repression domain (SRDX), which suppressed target genes of endogenous TCP3 and its functionally redundant TCPs. We found that the replum width of the *pAtTCP3::AtTCP3SRDX* siliques was similar to *jaw-D* siliques (Figure 1C). These results further suggested that miR319-regulated TCPs played critical role in replum development in siliques.

### 3.2. AtTCP3 Negatively Regulated Repla Development and Reduced the Lignification in Siliques

**Figure 3:**
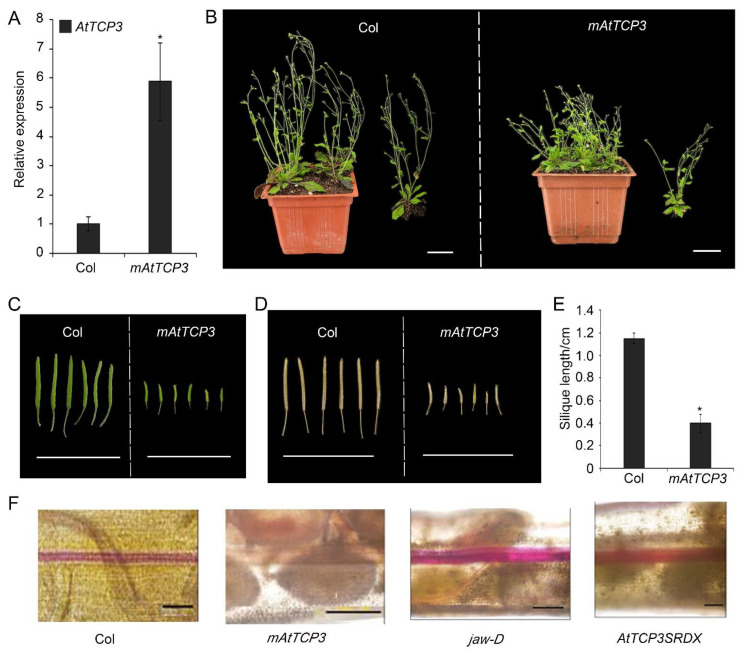
Silique phenotype and lignification of repla in *mAtTCP3* transgenic plants. (**A**) Real-time PCR showing expression levels of *AtTCP3* in *p35S::mAtTCP3* siliques. (**B**) Phenotype of the wild-type and *p35S::mAtTCP3* plants at reproductive stage. (**C**) Green siliques (stage 17) of the wild-type (left) and *p35S::mAtTCP3* (right) plants. (**D**) Mature siliques (stage 18) of the wild-type (left) and *p35S::mAtTCP3* (right) plants. (**E**) Graph showing silique length of the wild-type (Col) and *mAtTCP3* (*p35S::mAtTCP3*) plants. (**F**) Phloroglucinol staining showing lignin-specific signals in the siliques of Col, *jaw-D, mAtTCP3*, and *AtTCP3SRDX* plants. *mAtTCP3*, *p35S::mAtTCP3* lines. *AtTCP3SRDX*, *pAtTCP3:: AtTCP3SRDX*. The relative transcript level of each gene was normalized to *Actin* cDNA for quantification. Error bars indicate the standard errors. The asterisks indicate a significant difference (* *p* < 0.05). Scale bars in (**B**): 4cm. Scale bars in (**C**) and (**D**): 2 cm. Scale bars in (**F**): 5 mm.

To further study the function of TCP3 in developing siliques, a miR319a-resistant version (*p35S::mAtTCP3*) of *AtTCP3* was introduced into *Arabidopsis*. As expected, the *AtTCP3* transcription level was significantly increased in the transgenic plants compared with wild-type (Figure 3A). In wild-type siliques, valve margins or DZ were differentiated as a narrow strip consisted of few cells within the separation layer and the lignified regions, both of which contributed to the active fruit-opening process [19]. Consistent with the previous studies [43], *p35S:mAtTCP3* plants were smaller than wild-type plants (Figure 3B), and their siliques were much shorter (Figure 3C–E). Based on the SEM image of siliques (Stage 17), the repla of *p35S:mAtTCP3* siliques were much narrower than those in wide type and *jaw-D* siliques (Figure 1A). Cross sections in the middle of siliques also showed that the replum cells were relatively smaller in *p35S:mAtTCP3* siliques than in wild type and *jaw-D*. In addition, a layer of large and sparse cells were seen between the valve and the replum in the Col silique, which facilitated the dehiscence of the siliques, whereas this layer of cells was much denser in *p35S::mAtTCP3* siliques (Figure 1B). These results suggested that AtMIR319a-regulated TCP3 functioned as a negative regulator of silique development.

Silique wall thickness and replum development are correlated with lignin accumulation in plants [5]. To investigate whether TCP3 play a role in regulating replum lignification, phloroglucinol, a lignin-specific histological stain, was applied to siliques of wild-type, *p35S::mAtTCP3*, *pAtTCP3::AtTCP3SRDX*, and *jaw-D* plants for evaluating the degree of tissue lignification. In the wild-type siliques, lignin-specific signals were clearly seen in the repla and valve margin cells adjacent to the DZ throughout the siliques, while the signals were very faint in the repla and valve margin cells of *p35S::mAtTCP3* siliques (Figure 3F). In contrast, the lignin-specific signals were much stronger in the repla and valve margin cells of *jaw-D* and *pAtTCP3::AtTCP3SRDX* siliques compared to those in wide type. These results indicated that *AtTCP3* overexpression attenuated lignification of repla in the siliques.

### 3.3. AtFUL Acted Downstream of AtTCP3 in Replum Deficiency

**Figure 4:**
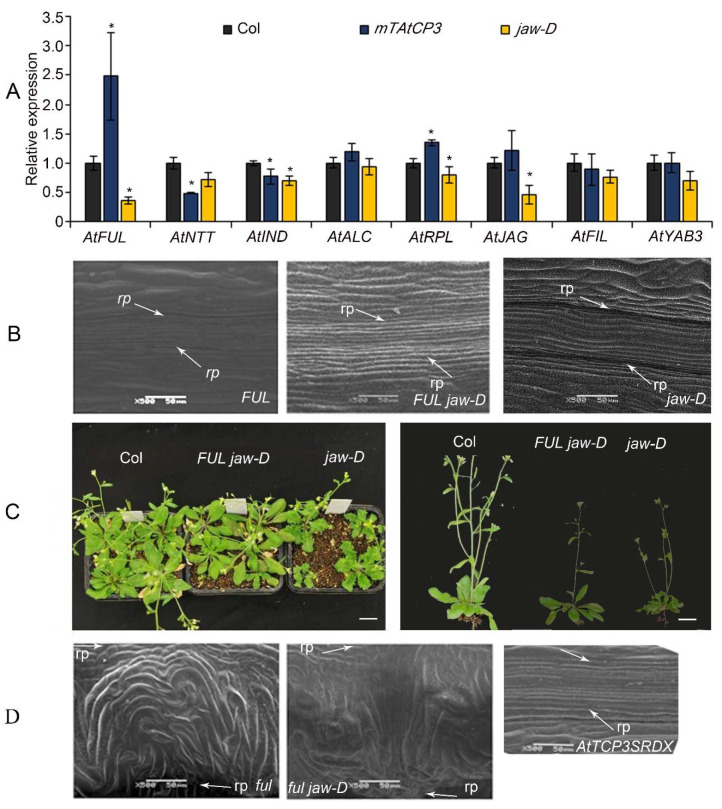
The relationship of miR319a and *AtTCP3* with replum- and valve-related genes. (**A**) Real-time PCR showing the relative expression levels of *AtFUL*, *AtNTT*, *AtIND*, *AtALC*, *AtRPL*, *AtJAG*, *AtFIL*, and *AtYAB3* in *p35S::mAtTCP3* and *jaw-D* siliques. (**B**) SEM images showing repla in the siliques of *FUL* and *FUL jaw-D* plants (*p35S::AtFUL* in *jaw-D* mutant). (**C**) The phenotypes of *FUL jaw-D* plants. (**D**) SEM images showing repla in siliques of *ful*, *ful jaw-D*, and *pAtTCP3::AtTCP3SRDX* plants. *AtTCP3SRDX* indicates *pAtTCP3:: AtTCP3SRDX*. The relative transcript level of each gene was normalized to *Actin* cDNA for quantification. Error bars indicate the standard errors. The asterisks indicate a significant difference (* *p* < 0.05).

Recent molecular and genetic studies in *Arabidopsis* have identified several crucial genes involved in the regulation of silique development [45]. To determine the relationship of TCP3 with those valve- and valve-margin-related genes, the expressional level of *AtFUL*, *AtNTT*, *AtIND*, *AtALC*, *AtJAG*, *AtFIL*, *AtYAB3*, and the replum-related gene *AtPRL*, were analyzed using real-time PCR. Transcript levels of *AtFUL* and *AtRPL* were significantly elevated in the *p35S::mAtTCP3* plants as compared to wild-type (Figure 4A). In addition, transcript levels of *AtFUL*, *AtIND*, *AtRPL,* and *AtJAG* were significantly downregulated in the *jaw-D* mutants compared to those in the wild type (Figure 4A). In combination, these results implied that *AtTCP3* acted as an upstream regulator of *AtFUL* to suppress the differentiation of the replum.

To further understand the genetic relationship between *AtTCP3* and *AtFUL*, the *AtFUL* gene were either overexpressed or suppressed in *jaw-D* mutant background. In *FUL jaw-D* plant overexpressing *FUL*, silique repla were narrower in width than that of *jaw-D* silique but still wider than the wild type, indicating that the wide replum phenotype of *jaw-D* mutant was partially complemented by *AtFUL* (Figure 1C and Figure 4B). Besides, the wavy leaf margins phenotypes observed in jaw-D were also restored by *AtFUL* overexpression in *FUL jaw-D* plants (Figure 4C). Moreover, *ful jaw-D* double mutants showed similar repla width with *ful* mutant (Figure 1C and Figure 4D). Together, these results suggest that *AtFUL* acted downstream of *TCP* genes.

### 3.4. Hyper-Activation of BnTCP3.A8 Affected Silique Development in Rapeseed

**Figure 5:**
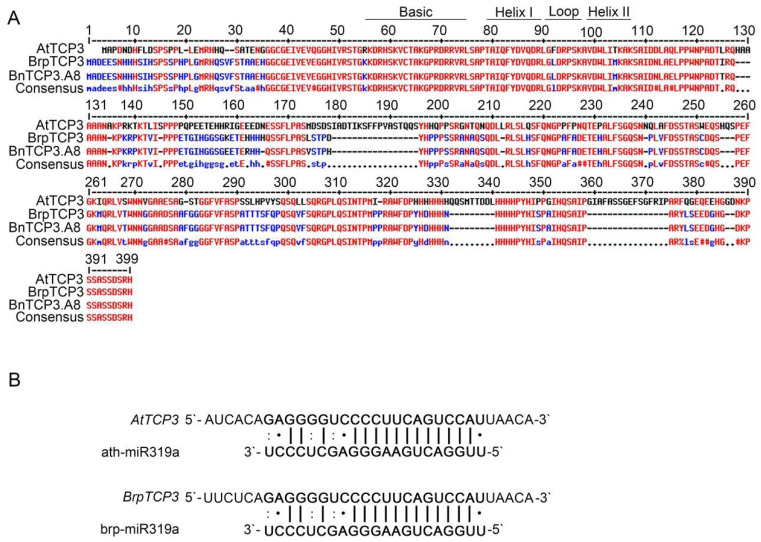
Alignment of putative TCP3 proteins in *Arabidopsis thaliana*, *B. rapa,* and *B. napus* and miR319a target sites of *AtTCP3* and *BrpTCP3*. (**A**) Amino acid sequence alignment of TCP3 proteins in *Arabidopsis*, *B. rapa*, and *B. napus.* The TCP domains are represented by black solid lines. (**B**) Reverse complementation between mature miR319a and *TCP3* mRNA.

Rapeseed and *Arabidopsis* are members of the Brassicaceae family and display similar silique morphologies. To investigate whether *TCP3* is also involved in replum development of rapeseed, we identified the *B. napus TCP3* gene by blast searching for the AtTCP3 protein coding sequence in the Brassica database (BRAD). The obtained locus, GSBRNA2T00114181001, which located on chromosome A08, showed the highest similarity to AtTCP3. Thus, this gene was named as *BnTCP3.A8* in following studies (Figure 5A, Figure S1A,B). Further sequence alignment of this gene in the same database revealed that BnTCP3.A8 displayed a 98% similarity to *B. rapa* BrpTCP3 (Figure 5A). *BnTCP3.A8* also contained a conserved BrpmiR319a-targeted site within the identified sequences (Figure 5B).

**Figure 6:**
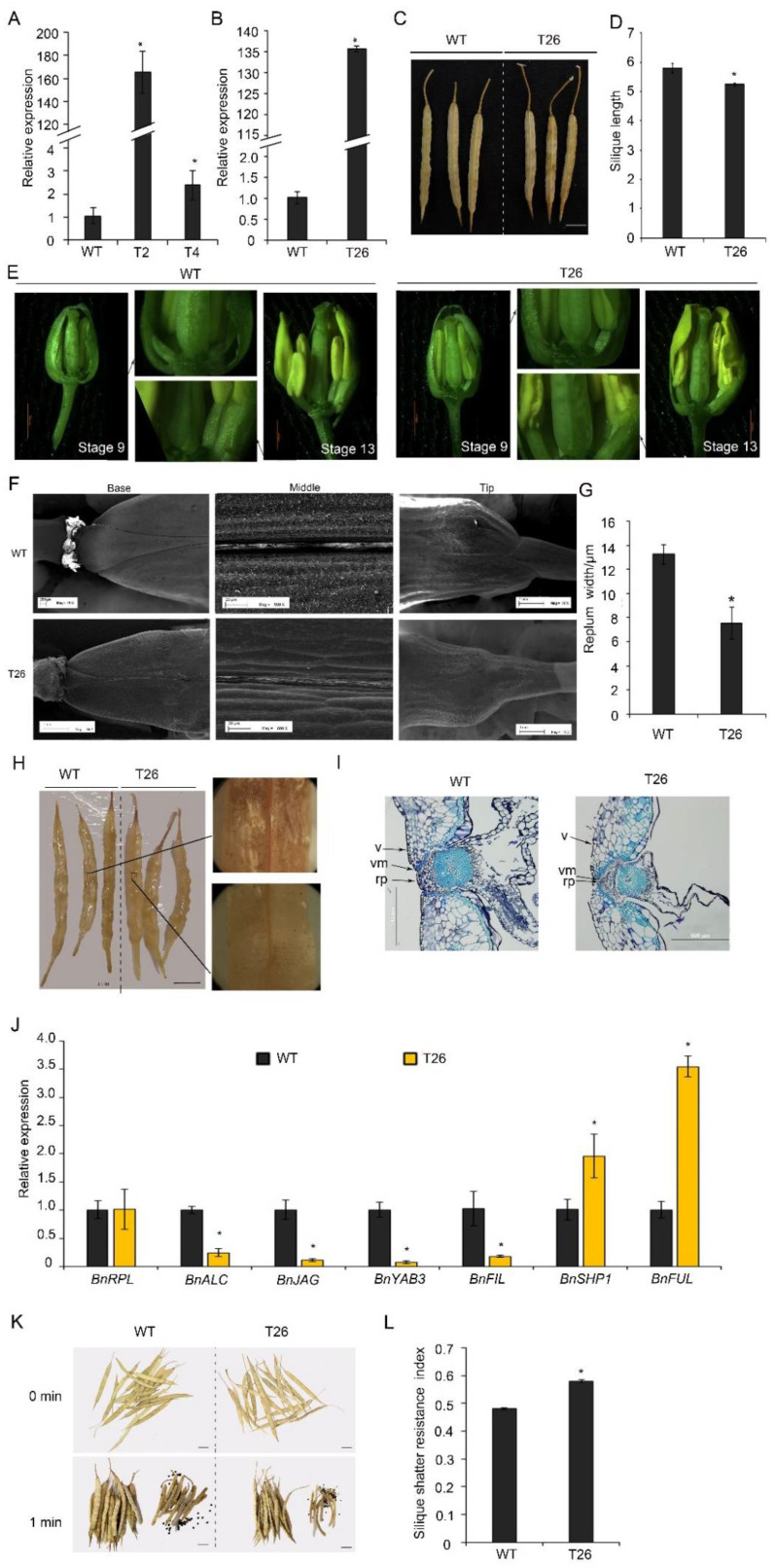
Silique shattering in rapeseed plants overexpressing *BnTCP3.A8*. (**A**) Real-time PCR showing the relative expression level of *BnTCP3.A8* in siliques (one week after pollination) of *BnTCP3.A8*-transgenic lines T2 and T4. (**B**) Real-time PCR showing the relative expression of *BnTCP3.A8* in the siliques (one week after pollination) of *BnTCP3.A8*-transgenic transgenic line T26. (**C**) The siliques at the pre-harvesting stage. (**D**) Graph showing silique length of the wild-type and *BnTCP3.A8*-transgenic lines T26. (**E**) The phenotypes of *BnTCP3.A8*-transgenic lines T26. At stage 9, the magnified views of silique (right) are shown. At stage 13, the magnified views of silique (left) are shown. (**F**) SEM images showing repla in siliques of the transgenic line T26. (**G**) Replum width of *BnTCP3.A8*-transgenic line T26. (**H**) Phloroglucinol staining of the surfaces of siliques in the transgenic line T26. Magnified views of silique (right) are shown. (**I)** Cross-sections of WT and T26 siliques showing repla and valve margins. (**J**) Real-time PCR showing the relative expression levels of *BnRPL*, *BnALC*, *BnJAG*, *BnYAB3*, *BnFIL*, *BnSHP1*, and *BnFUL* in the siliques of the transgenic line T26. The relative transcript level of each gene was normalized to *UBC21* cDNA for quantification. (**K**) One min after silique shattering treatments. Seeds from shattered siliques after agitation in the random impact test. (**L**) Silique shattering resistance index of *BnTCP3.A8*-transgenic line T26. The proportions of siliques opened versus agitation time in the random impact test. Error bars indicate the standard errors. The asterisks indicate a significant difference (*p <* 0.05). Scale bars in (**C**), (**H**,**K**) 1 cm. WT, accession K407. T26, *p35S::TCP3.A8* line. rp, replum. v, valve. vm, valve margin. * indicates *p* < 0.05.

To study the functions of *BnTCP3.A8* in silique development, the *BnTCP3.A8* CDS was introduced into rapeseed plants (accession K407) under the regulation of the cauliflower mosaic virus 35S promoter. The expression of *BnTCP3.A8* was significantly upregulated in *p35S::BnTCP3.A8* transgenic lines T2, T4 (T2 generation), and T26 (T3 generation) (Figure 6A,B). In those plants, silique lengths of the *p35S::BnTCP3.A8* line were shorter than that of the wild-type (Figure 6C,D). For immature gynoecium [46], the placenta development of *p35S::BnTCP3.A8* line was weaker than that of the wild-type (Figure 6E). Further, the image of SEM showed that the replum in T26 silique was much narrower than that of the wild-type (Figure 6F). We measured the replum width in the middle of siliques. Replum width of T26 silique was significantly narrower than that of the wild-type (Figure 6G). These results indicated that *BnTCP3.A8* negatively regulated replum development in rapeseed, consistent with the role of *AtTCP3* in *Arabidopsis*.

Spence et al. (1996) showed that silique shattering resistance was negatively associated with the degree of lignification in the valves, repla, and valve margin cells [18]. To investigate whether the *p35S::BnTCP3.A8* plants also reduced lignification in repla, the siliques of both the wild-type and transgenic lines were stained by phloroglucinol. The siliques of T26 showed weaker lignin-specific signals in the repla than those of the wild-type (Figure 6H). In addition, the siliques of T26 exhibited smaller valve margin cells adjacent to the DZs (Figure 6I). These observations indicated that the reduced lignification in the repla was responsible for the resistance of *p35S::BnTCP3.A8* plants to silique shattering.

### 3.5. Hyper-Expression of BnTCP3.A8 Upregulated BnFUL-BnSHP1 Network and Enhanced Silique Shattering Resistance in Rapeseed

To determine whether *BnTCP3.A8* is also involved in the regulation of replum- and valve-related genes as *AtTCP3*, we analyzed expression levels of *BnFUL* and *BnSHP1, BnALC*, *BnJAG*, *BnFIL*, and *BnYAB3* genes in T26 siliques. Real-time PCR showed that *BnFUL* and *BnSHP1* were upregulated in *p35S::BnTCP3.A8* siliques, while *BnALC*, *BnJAG*, *BnFIL*, and *BnYAB3* were downregulated (Figure 6J). These results revealed that the *TCP3*-mediated gene regulatory pathways were conserved in rapeseed during silique development.

To define whether the deficiency in replum width of rapeseed affects silique shattering, we determined the silique shattering ratios using the modified method of Bruce et al. (2002) [47]. The siliques of the wild-type plants were opened easily by shattering treatment and released many seeds (Figure 6K); however, the siliques of T26 plants were less opened than the wild-type and released fewer seeds. The SRI (shattering-resistance index) of T26 was 0.58 at 300 rpm, which was significantly high than the SRI of wild-type (SRI = 0.48) (Figure 6L). Thus, these results suggested that overexpression of *BnTCP3.A8* enhanced silique shattering resistance.

## 4. Discussion

### 4.1. miR319-Targeted TCP Genes Inhibited Replum Enlargement

The miR319-targeted *TCP* genes control cell division arrest [25,27,28,48,49,50]. In general, proteins encoded by genes expressed in the replum often negatively regulate genes expressed in the valves [1]. We found that these *TCP* genes were involved in development of repla because repla became wider in *jaw-D* mutant. Among them, *AtTCP3* was down-regulated mostly among these *TCP* genes. In situ hybridization revealed that *AtTCP3* was preferentially expressed in middle region of carpel. In addition, *p35S::mAtTCP3* reduced the replum width, and *pTCP3::mAtTCP3SRDX* increased the replum width. Taken together, these results suggested that *AtTCP3* functioned in regulation of replum development.

The factors that control the establishment of medio-lateral silique patterns also regulate proper shoot development and leaf formation [51,52,53,54,55]. We found that *AtTCP3* positively regulated valve- and valve margin-related genes *AtFUL* and *AtSHP1*. Genetic analysis showed *AtFUL* acted downstream of miR319a-targeted *AtTCP3* in replum deficiency. This evidence indicates that *TCP3* functioned in repressing replum enlargement.

### 4.2. BnTCP3.A8 Enhanced Silique Shattering Resistance

Cell wall lignification is a complex process that only occurs in higher plants, and its main function is to strengthen plant vascular bodies. Cell wall lignification affects silique dehiscence in siliques. *SHP1* and *SHP2* promotes valve margin lignification [8]. In this study, we found that the lignin-specific staining of the repla of *Arabidopsis* *p35S::mAtTCP3* and *p35S::BnTCP3.A8* plants was weaker as compared to wild-type, while the lignin-specific staining of the repla of *jaw-D*, and *pAtTCP3::AtTCP3SRDX* siliques were much stronger than that of the wild type.

The premature cracking of siliques before or during ripening causes silique shattering and drastically reduces production. Rapeseed is widely planted in temperate regions. Total yields may reduce 20% owing to silique shattering, and in arid environments this reduction reaches 50% [56,57]. In recent years, some progress has been made in transgenic approaches using *Arabidopsis* genes to produce indehiscence rape [58]. Chung et al. (2013) reported that the *ntt-3D* mutant, an activation tagged allele of *NTT*, showed an enlarged replum and the fruit indehiscence in *Arabidopsis* [13]. Overexpression of *AtFUL* gene in *B. juncea* produces pod shatter-resistant *Brassica* fruit [19]. The shatter-resistant *B. juncea* siliques may have smaller non-lignified separation layers than *B. napus* siliques [59]. A similar phenotype was observed in *35S::FUL Arabidopsis* plants, which showed reductions in the lignification of cells adjacent to the DZs, resulting in the formation of indehiscent siliques that did not release seeds normally [20]. The replum of the transgenic line *p35S::BnTCP3.A8* displayed a narrower replum and a lower degree of lignification, compared with that of the wild type, and t *p35S::BnTCP3.A8* plants exhibited higher silique shatter resistance. Thus, the deficiency in replum may affect silique shattering. Whether are the size and shape of valve margin changed by *TCP3* deregulation remains unclear. Both the *Arabidopsis p35S::mAtTCP3* and *B. napus p35S::BnTCP3.A8* plants exhibited shorter and smaller siliques. This fact implies that the effects of *TCP3* on silique development are much broader than expected. An attempt to explore the new function of *TCP3* and the other miR319a-targeted gene is underway in our lab. In *FUL jaw-D* double mutants, silique repla were narrower in width than that of *jaw-D* silique but still wider than the wild-type, and *ful jaw-D* double mutants no significant change of repla width was observed compared to *ful* mutant. *TCP3* positively regulated *FUL* expression and affected replum lignification in *Arabidopsis* and *B. napus*. Alternatively, *TCP3* may inhibit replum growth in a direct or indirect path unknown. Our results provide novel insights into the mechanisms of silique dehiscence in *Arabidopsis* and *B. napus*, which can most likely be applied to other crops and lay a foundation for the development of oil crop varieties having strong shattering-resistance levels.

## 5. Conclusions

In Summary, this study revealed a critical role of miR319-regulated *TCPs* in silique development, reducing replum width and lignification via FUL-regulated pathway, which contributed to the silique shattering resistance in both *Arabidopsis* and *B. napus*. Specifically, we found that hyper-activation of *BnTCP3.A8* enhanced silique resistance to shattering in rapeseed, providing a potential genetic locus for molecular breeders to improve silique shattering resistance in Brassica crops.

## Data Availability

Not applicable.

## Supplementary Materials

The following supporting information can be downloaded at: https://www.mdpi.com/article/10.3390/cells11193096/s1, Table S1. Primer and probe sequences used in this study; Figure S1. Alignment and phylogenetic analysis of miR319-regulated TCP proteins in *Arabidopsis* and TCP3 homologous proteins in *B. rapa* and *B. napus*. (**A**) Alignment of the targets of miR319 in *Arabidopsis* with TCP3 proteins in *B. rapa* and *B. napus*. (**B**) Phylogenetic tree analysis of miR319-regulated TCP proteins in *Arabidopsis* and TCP3 homologus protein in *B. rapa* and *B. napus*.
