# Supplementary material for: miR319-Regulated TCP3 Modulates Silique Development Associated with Seed Shattering in Brassicaceae
Source: Cells. 2022 Oct 1;11(19):3096. doi: 10.3390/cells11193096 (PMC9563637; doi:10.3390/cells11193096)
Supplement: Supplementary file 1 [file cells-11-03096-s001.zip › Supplementary Figure S1.pdf]

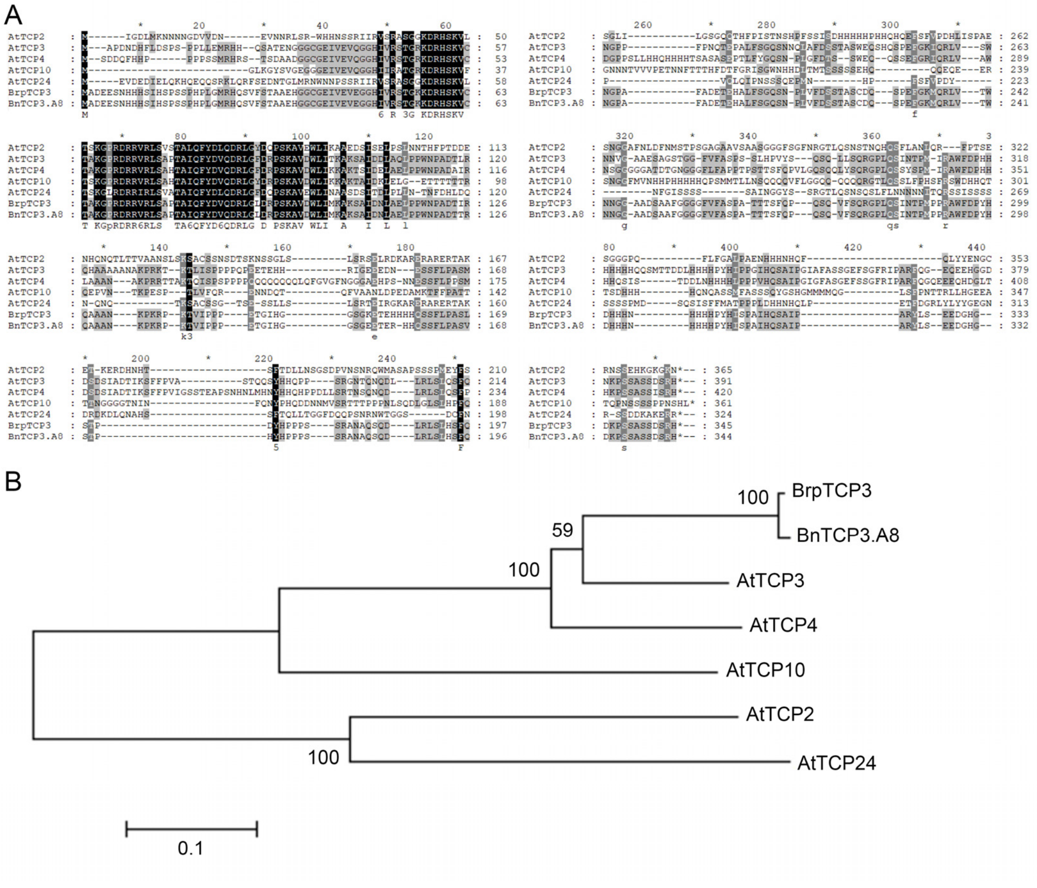

Figure S1. Alignment and phylogenetic analysis of miR319-regulated TCP proteins in *Arabidopsis* and TCP3 homologous proteins in *B. rapa* and *B. napus*. (**A**) Alignment of the targets of miR319 in *Arabidopsis* with TCP3 proteins in *B. rapa* and *B. napus*. (**B**) Phylogenetic tree analysis of miR319-regulated TCP proteins in *Arabidopsis* and TCP3 homologus protein in *B. rapa* and *B. napus*.
